# Low Levels of DNA Polymerase Alpha Induce Mitotic and Meiotic Instability in the Ribosomal DNA Gene Cluster of *Saccharomyces cerevisiae*

**DOI:** 10.1371/journal.pgen.1000105

**Published:** 2008-06-27

**Authors:** Anne M. Casper, Piotr A. Mieczkowski, Malgorzata Gawel, Thomas D. Petes

**Affiliations:** Department of Molecular Genetics and Microbiology, Duke University Medical Center, Durham, North Carolina, United States of America; Stowers Institute for Medical Research, United States of America

## Abstract

The ribosomal DNA (rDNA) genes of *Saccharomyces cerevisiae* are located in a tandem array of about 150 repeats. Using a diploid with markers flanking and within the rDNA array, we showed that low levels of DNA polymerase alpha elevate recombination between both homologues and sister chromatids, about five-fold in mitotic cells and 30-fold in meiotic cells. This stimulation is independent of Fob1p, a protein required for the programmed replication fork block (RFB) in the rDNA. We observed that the *fob1* mutation alone significantly increased meiotic, but not mitotic, rDNA recombination, suggesting a meiosis-specific role for this protein. We found that meiotic cells with low polymerase alpha had decreased Sir2p binding and increased Spo11p-catalyzed double-strand DNA breaks in the rDNA. Furthermore, meiotic crossover interference in the rDNA is absent. These results suggest that the hyper-Rec phenotypes resulting from low levels of DNA polymerase alpha in mitosis and meiosis reflect two fundamentally different mechanisms: the increased mitotic recombination is likely due to increased double-strand DNA breaks (DSBs) resulting from Fob1p-independent stalled replication forks, whereas the hyper-Rec meiotic phenotype results from increased levels of Spo11-catalyzed DSBs in the rDNA.

## Author Summary

In many organisms, the genes that encode the ribosomal RNAs are present in multiple copies arranged in tandem. This unique section of the genome is under strict cellular control to minimize changes in the number of ribosomal DNA (rDNA) genes as a consequence of unequal crossover between repeats. In addition, the rate of unequal crossovers and gene conversion in the rDNA influence the level of sequence divergence between repeats. Crossovers can result from repair processes initiated at stalled replication forks, and in this study we investigated the effect of a low level of DNA polymerase on rDNA stability. We found that low levels of DNA polymerase modestly increase rDNA recombination in mitosis and strongly elevate rDNA recombination in meiosis.

We suggest that in mitotic cells the increased recombination is likely due to increased double strand DNA breaks (DSBs) resulting from stalled replication forks. However, in meiotic cells, we found evidence that the high level of recombination results from increased levels of Spo11-catalyzed DSBs in the rDNA. Our results indicate that there are two fundamentally different mechanisms in mitosis and meiosis for the maintenance of rDNA stability.

## Introduction

The maintenance of genetic stability during DNA replication is of critical importance. DNA polymerases can stall at DNA lesions such as crosslinks, strand breaks, natural pause sites, and regions that can form secondary structures [1],[2]. Stalled replication forks are a potential source of genetic instability, because they can be processed to a double-strand break (DSB) [3],[4]. Recombination proteins form foci at stalled forks, and homologous recombination (HR) is thought to be one mechanism by which collapsed forks are re-initiated [5],[6].

Almost 10% of the *S. cerevisiae* genome is within the rDNA array, a cluster of 150–200 tandemly repeated 9 kb units on the right arm of chromosome XII [7],[8]. Each 9 kb unit has a natural replication fork barrier (RFB) site. The RFB prevents replication fork progression in the direction opposite 35S transcription, presumably to prevent collisions between DNA and RNA polymerases [9]–11. The Fob1p binds directly to the RFB sequence and is required for replication fork blocking [12]. Double-strand breaks (DSBs) are observed near the RFB site in logarithmically growing cells [13],[14] and are a source of genetic instability within the array, leading to high levels of unequal sister-chromatid exchange, unequal gene conversion, and intra-chromatid recombination [15]–[18]. Cells that lack the Fob1p do not experience fork stalling at the RFB and have reduced mitotic rDNA recombination [14], [17]–[19]. In these studies, the effect of the *fob1* mutation on rDNA recombination between homologues was not examined.

In contrast to the relatively high levels of mitotic recombination in the rDNA, meiotic recombination between rDNA arrays on homologous chromosomes is suppressed 70- to 100-fold [8],[20]. The mechanism preventing meiotic rDNA recombination between homologs is not yet fully understood. Meiosis-specific DSBs are undetectable in the array [21], and Spo11p, which catalyzes meiotic DSBs, is at low levels within the array [22]. Strains that lack Sir2p have increased Spo11p-associated DSBs in the rDNA [22] and significantly elevated meiotic and mitotic unequal sister-chromatid rDNA recombination [22]–[24].

In this study, we designed a system that allows us to measure rDNA recombination both between homologues and between sister chromatids. Using this system, we examined the relationship between DNA replication and recombination by investigating mitotic and meiotic rDNA recombination in cells with low levels of Pol1p, the catalytic subunit of the lagging strand DNA polymerase alpha [25]. Reduced levels of Pol1p were previously shown to elevate the rates of translocations, chromosome loss events, microsatellite alterations, deletions and point mutations in non-rDNA regions [26],[27]. Below, we show that low levels of Pol1p significantly increase recombination in the rDNA array, both between homologues and between sister chromatids. This increase is observed in both mitosis and meiosis. These data suggest that the mechanisms controlling rDNA recombination are closely coordinated with the replication machinery.

## Results

### Low Levels of Pol1p Increase Mitotic rDNA Recombination

**Figure 1:**
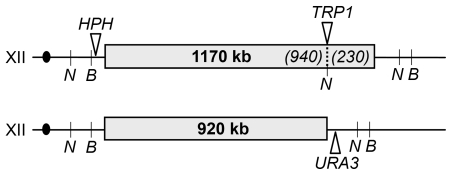
Positions of heterozygous markers used to monitor rDNA recombination in diploids. Grey boxes indicate the rDNA array and the black circles show the centromeres. Chromosome XII sequences surrounding the array are not drawn to scale. Positions of *Bam*HI (*B*) and *Ngo*MIV (*N*) restriction sites are indicated. *Bam*HI has no sites within the rDNA or within the *TRP1* insertion, whereas *Ngo*MIV has no sites within the rDNA but a site within the *TRP1* gene. Using CHEF gel analysis of *Bam*HI- or *Ngo*MIV-treated DNA samples, we determined the sizes of the rDNA clusters and the position of insertion of *TRP1* within the clusters. We depict the location of the markers in the wild-type diploid AMC45. The sizes of the *HPH*-containing cluster, the positions of the *TRP1* marker within the cluster (from the centromere-proximal junction), and the sizes of the *URA3*-containing clusters in kb for the other strains are, respectively: 1080, 880, 1110 (*GAL-POL1* strain, AMC20); 1170, 940, 920 (*fob1* strain, AMC156); 960, 580, 1110 (*GAL-POL1 fob1* strain, AMC160).

In order to investigate the effect of low Pol1p levels on rDNA instability, we used a diploid strain homozygous for the *GAL-POL1* allele, in which the *POL1* gene is fused to the *GAL1/10* promoter [26]. Low galactose levels (0.005%) induce Pol1p expression at ∼10% of the wild-type level, whereas high galactose (0.05%) induces ∼300% expression of Pol1p compared to wild-type. For our analysis, we constructed strains containing heterozygous markers surrounding and within the rDNA array. On one homologue, we inserted the *URA3* gene at the centromere-distal junction of the rDNA. On the other homologue, we inserted the *HPH* gene (encoding hygromycin resistance) centromere-proximal to the rDNA, and a copy of *TRP1* within the rDNA array (Figure 1). The rDNA cluster sizes in these strains were determined by clamped homogenous electric field (CHEF) gel electrophoresis of genomic DNA digested with *Bam*HI which does not cut within the array or the *TRP1* insertion. The location of *TRP1* within the array was determined by digestion with *NgoMIV* that cuts in *TRP1* but not within the rDNA (Figure 1).

**Figure 2:**
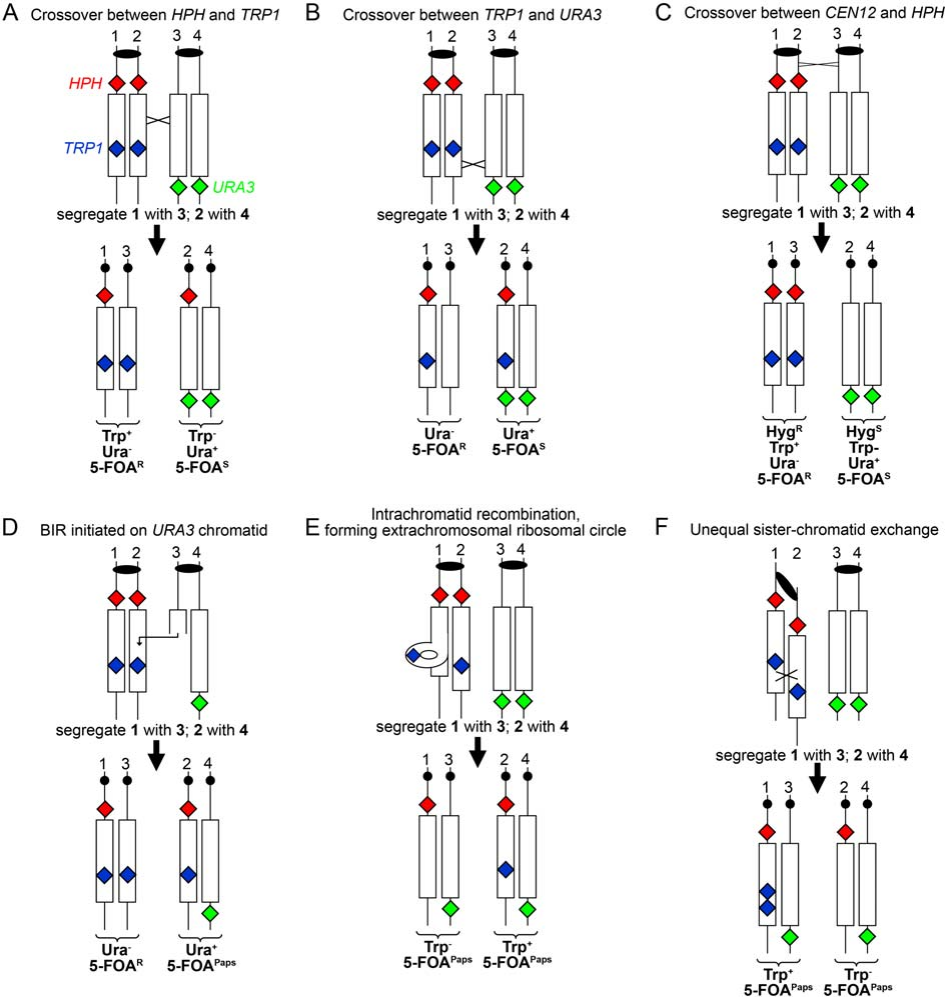
Phenotypic classes of sectored colonies resulting from crossover between homologues and inter- or intra-sister chromatid recombination in the rDNA array. Cells with the arrangement of markers shown in Figure 1 were plated on rich growth medium and then replica-plated to medium lacking tryptophan or uracil or containing hygromycin. (A) Reciprocal crossover (RCO) between *HPH* and *TRP1* results in one Trp^+^, Ura^−^ (5-FOA^R^) cell and one Trp^−^, Ura^+^ (5-FOA^S^) cell. (B) RCO between *TRP1* and *URA3* results in one Ura^−^ (5-FOA^R^) and one Ura^+^ (5-FOA^S^) cell. (C) RCO between *CEN12* and *HPH* results in sectoring for all three markers. (D) A break-induced replication (BIR) event initiated by a DSB within the rDNA of the *URA3*-containing chromosome or gene conversion will result in a Ura^+^/Ura^−^ sectored colony. Cells derived from the Ura^+^ part of the sector will form 5-FOA^R^ papillae (5-FOA^Paps^) as a consequence of secondary recombination events that cause loss of *URA3*. (E) Intra-chromatid recombination events that span the *TRP1* marker will result in a Trp^+^/Trp^−^ sectored colony, both sectors having the 5-FOA^Paps^ phenotype. The same pattern will be observed if the *TRP1* marker is lost by gene conversion or single-strand annealing. (F). Unequal sister-chromatid exchange. As in Figure 2E, this event will result in a Trp^+^/Trp^−^ sectored colony, both sectors having the 5-FOA^Paps^ phenotype. The cells in the Trp^+^ sector, however, have two copies of the *TRP1* insertion rather than one.

To measure the rate of mitotic rDNA recombination, we incubated *GAL-POL1* diploids in media containing high or low galactose levels for six hours, followed by plating onto medium with high galactose. Wild-type diploids were grown in rich growth medium media for six hours, followed by plating on rich growth medium. Colonies formed on the plates were replica plated to media lacking uracil or tryptophan, or containing hygromycin. Cells undergoing a recombination event within the rDNA at the time of plating will appear as sectored colonies on the diagnostic media. Using the phenotypes of the sectors, we can diagnose reciprocal crossovers (RCOs; Figure 2A–C) as well as various other types of recombination (Figure 2D–F). We can only detect half of RCO events, because only one of the two possible chromosome segregation patterns will produce a sectored colony. Since these two patterns of segregation are equally frequent [28], we calculate that the rate of RCO is twice the frequency of sectored colonies. Non-reciprocal recombination events (Break-Induced Replication [BIR] [29]) can result in a loss of one marker (for example, *URA3* as shown in Figure 2D) and duplication of another (*TRP1*). Loss of the *TRP1* marker by intrachromatid “pop-out” exchange (Figure 2E), single-strand annealing (not depicted), or unequal sister-chromatid exchange (Figure 2F) can also be detected.

**Figure 3:**
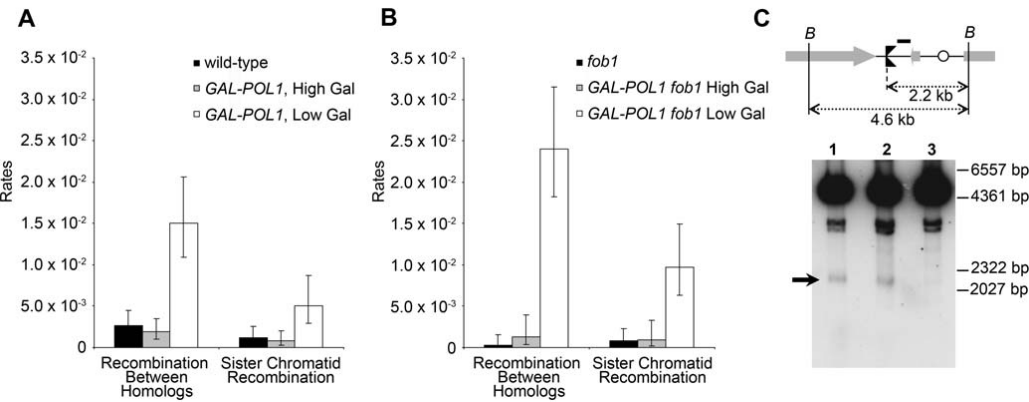
Comparison of mitotic rDNA recombination rates and DSBs near the RFB. (A) Rates of mitotic rDNA recombination between homologues and sister chromatids in wild-type cells (rich growth medium), and *GAL-POL1* cells grown on media with either high or low galactose levels. Recombination between homologues is the sum of all RCO and BIR categories (Table 1). 95% confidence intervals are shown. (B) Rates of mitotic rDNA recombination in *fob1* cells (rich growth medium) and *GAL-POL1 fob1* cells grown on media with either high or low galactose levels. (C) DSBs near the RFB in wild-type cells (lane 1), *GAL-POL1* cells grown in low galactose (lane 2), and *fob1* cells (lane 3). In the diagram, the key is: 35S rRNA (large gray arrows), 5S rRNA (small grey arrow), RFB (black double triangles), replication origins (open circles), and *Bgl*II sites (*B*). DNA was isolated, and treated with *Bgl*II. The resulting fragments were examined by Southern analysis, using rDNA probe #1 (shown as short horizontal bar). The 2.2 kb fragment representing the DSB at the RFB (marked with a black arrow) is evident in lanes 1 and 2, but not in 3.

To analyze further the type of recombination event responsible for sectoring, we did several types of analysis. First, we purified all sectored colonies and, in colonies with Ura^+^/Ura^−^ sectors, we determined whether the Ura^+^ cells had a high rate of 5-fluoro-orotate-resistant (5-FOA^R^) derivatives (indicating that the cells were heterozygous for the *URA3* insertion) or had a very low rate of 5-FOA^R^ derivatives (indicating that the cells were homozygous for the insertion). In strains heterozygous for the insertion, a 5-FOA^R^ derivative could arise by loss of the wild-type *URA3* allele by a subsequent mitotic crossover or by chromosome loss. We also subjected each sector side of the colony to tetrad analysis. Some Trp^+^/Trp^−^ sectored colonies were further analyzed by Southern analysis to determine whether sectoring arose by unequal crossover, intrachromatid recombination, or other events; this analysis is discussed in detail in Text S1. Based on this analysis (summarized in Table 1), we grouped these mitotic events into two categories: rDNA recombination between homologues and between sister chromatids. In cells with low levels of Pol1p (Figure 3A), we observed a four- to five-fold increase relative to wild-type cells or cells with high levels of Pol1p in both of these categories (*p*<.0001 and *p* = 0.0031, respectively).

**Table 1:** Frequencies of sectored colonies of various phenotypes reflecting mitotic recombination in a wild-type strain and in strains with high and low levels of DNA polymerase alpha.

		rDNA recombination between homologues						
Genotype, growth condition, and number of colonies	*CEN12-HPH* RCO	*HPH-TRP1* RCO	*TRP1*-*URA3* RCO	BIR initiated on *URA3* chromatid	Gene conversion	BIR initiated on *HPH* chromatid	Total	Sister chromatid recombination
**wild-type**, n = 5197 colonies	1 (3.8×10^−4^)	5 (1.9×10^−3^)	0	2 (3.8×10^−4^)	1 (1.9×10^−4^)	1 (1.9×10^−4^)	**2.7×10^−3^**	6 (1.2×10^−3^)
***GAL-POL1,*** **High Gal**, n = 5156 colonies	0	2 (7.8×10^−4^)	1 (3.9×10^−4^)	1 (1.9×10^−4^)	2 (3.9×10^−4^)	1 (1.9×10^−4^)	**1.9×10^−3^**	4 (7.8×10^−4^)
***GAL-POL1***, **Low Gal**, n = 2393 colonies	3 (2.5×10^−3^)	12 (1.0×10^−2^)	5 (4.2×10^−3^)	0	0	1 (4.2×10^−4^)	**1.5×10^−2^**	12 (5.0×10^−3^)

Following growth of cells in medium containing glucose (AMC45), or raffinose with 0.05% or 0.005% galactose (AMC20), cells were plated on glucose-containing medium (AMC45) or raffinose with 0.05% galactose (AMC20) and allowed to form colonies. These colonies were then replica-plated to medium lacking uracil or tryptophan, or containing hygromycin. The first number in each column is the number of colonies of each sectoring class. The number in parentheses represent the rate of the event. Since we can detect only one half of the reciprocal crossover (RCO) events, the frequency of sectored colonies was doubled to estimate the rate of RCOs (see text for details). The “Total” column shows the total rate of all recombination events occurring between homologues within the rDNA. Some patterns of sectoring are unambiguous in defining the type and position of the exchange. Other patterns are ambiguous unless further tests are performed. Sectored colonies in which one sector is Hyg^R^ Trp^+^ Ura^−^ and the other sector is is Hyg^S^ Trp^−^ Ura^+^ indicate reciprocal crossovers (RCOs) between the *HPH* marker and *CEN12* (Figure 2C). An RCO between the *HPH* and *TRP1* markers results in Trp^+^ Ura^−^ and Trp^−^ Ura^+^ sectors (Figure 2A). A RCO between *TRP1* and *URA3* results in Ura^+^/Ura^−^ sectors (Figure 2B); tetrad dissection of the Ura^+^ sector results in four Ura^+^ spores. A break-induced replication (BIR) event initiated on the *URA3*-containing chromatid between *HPH* and *TRP1* results in Ura^+^/Ura^−^ sectors (Figure 2D); tetrad dissection of the Ura^+^ sector results in 2∶2 segregation for both the *TRP1* and *URA3* markers, whereas dissection of the Ura^−^ sector results in 4^+^∶0^−^ segregation for the *TRP1* marker. In the colonies in the “Gene conversion” class, the colony has Ura^+^/Ura^−^ sectors. By tetrad dissection, the Ura^+^ sector yield two Ura^+^ and two Ura^−^ spores. The conversion event could be local (deleting only the *URA3* insertion) or represent a BIR event that occurs distal to the *TRP1* insertion. A BIR event initiated by a break on the *HPH*-containing chromatid results in a Trp^+^/Trp^−^ sectored colony; dissection of the Trp^+^ results in 2∶2 segregation of the *TRP1* marker. The category of sister-chromatid recombination includes formation of extrachromosomal circles (Figure 2E), unequal sister-chromatid exchange (Figure 2F), and gene conversion of the *TRP1* marker. We performed a physical analysis (described in Text S1) that indicated that, in six of nine colonies with Trp^+^/Trp^−^ sectors in the AMY20 strain, the Trp^+^ cells had two copies of the *TRP1* insertion, as expected from unequal sister-strand exchange.

### Increased Mitotic rDNA Recombination in Cells with Reduced Pol1p Is Not Fob1p-Dependent

To determine whether forks blocked at the RFB are the primary source of rDNA instability in cells with low Pol1p, we analyzed mitotic recombination rates in *fob1* mutant derivatives of our strains. Previous studies reported decreased extrachromosomal rDNA circle (ERC) formation and an approximately three-fold reduction in internal rDNA marker loss in *fob1* mutants [14], [17]–[19], presumably due to the decrease in recombinogenic DSBs at the RFB. We found that the *fob1* mutation alone resulted in a 10-fold reduction, relative to wild-type, in the rate of mitotic rDNA recombination between homologs (*p* = 0.011), but a statistically insignificant (*p* = .671) reduction in sister chromatid recombination (Figure 3B). Surprisingly, we showed that the *fob1* mutation resulted in a significant *increase* in recombination between homologues (*p* = 0.027) in the *GAL-POL1* strain grown on low galactose compared to the level observed in the *FOB1 GAL-POL1* strain grown under the same conditions. The classes of mitotic recombination events in strains with the *fob1* mutation (Table S1) were similar to those shown in Table 1. Thus, the hyper-Rec phenotype associated with low levels of DNA polymerase alpha does not reflect elevated levels of Fob1p-mediated stalling at the RFB.

We also directly investigated the level of DSBs at the RFB in our cells by Southern analysis (Figure 3C). In *Bgl*II-treated DNA from wild-type cells, the DSB associated with RFB (indicated by an arrow) is observed as a 2.2 kb fragment hybridizing to an rDNA probe (lane 1, Figure 3C), as reported previously [13],[14]. The amount of this fragment (normalized to the 4.6 kb unbroken *Bgl*II fragment) was about the same in the *GAL-POL1* strain with low levels of DNA polymerase alpha (lane 2, Figure 3C) as in the wild-type strain. The *fob1* mutation, as expected, reduced the amount of the 2.2 kb fragment (lane 3, Figure 3C). The pair of 3.0–3.5 kb bands observed in all samples have been observed previously [13],[14] and represent either a Fob1p-independent DSB in the rDNA or junction fragments of non-rDNA with rDNA.

### Alteration in the Size of the rDNA Gene Cluster Induced in Cells with Low Pol1p

**Figure 4:**
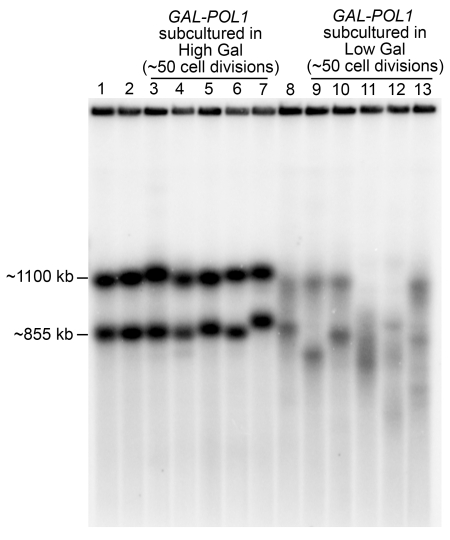
CHEF gel analysis of the size of rDNA arrays in cells with high and low levels of polymerase alpha. Individual colonies of a *GAL-POL1* strain were sub-cultured twice (about 50 cell divisions) in media with high or low levels of galactose. We subsequently isolated DNA from these derivatives in agarose plugs, treated the plugs with *Bam*HI (which does not cleave in the rDNA), and separated the resulting fragments by CHEF gel electrophoresis. The separated DNA molecules were transferred to membranes and hybridized to an rDNA-specific probe. The derivatives in each lane were: 1 (*GAL-POL1* strain before sub-culturing), 2 (*GAL-POL1* derivative sub-cultured once on high galactose), 3–7 (five individual derivatives sub-cultured twice on high galactose), 8 (*GAL-POL1* strain sub-cultured once on low galactose), 9–13 (five individual isolates sub-cultured twice on low galactose).

Unequal sister-chromatid recombination will result in loss of the *TRP1* marker only if the crossover occurs between the misaligned insertions (Figure 2F). In contrast, all unequal crossovers or intrachromatid crossovers that alter the size of the rDNA array by 50 kb or more can be detected by CHEF gel electrophoresis of *Bam*HI-treated DNA samples. To determine whether low DNA polymerase alpha resulted in increased size variability of the rDNA, we examined the sizes of rDNA clusters in sub-cultured isolates of the *GAL-POL1* diploid grown on plates containing high or low levels of galactose (Figure 4). The rDNA clusters in the initial *GAL-POL1* diploid colony were about 1100 kb and 855 kb. We observed slight size variation following two cycles of high galactose subculturing (lanes 3–7). The size variation in cultures sub-cultured in low galactose (lanes 9–13) was considerably greater, and two of the five colonies had three rDNA clusters, reflecting either chromosome XII trisomy or the presence of sub-populations within the culture with varying array sizes.

The rDNA bands derived from strains subcultured on low galactose were blurry in comparison to those subcultured in high galactose. It is likely that this blurring reflects a very high rate of recombination resulting in small changes in cluster size. We also observed that, in DNA samples isolated from the *GAL-POL1* cells in exponential phase, most of the chromosome XII DNA molecules were retained in the well of the gel rather than migrating in the normal position (Figure S1). Since branched DNA molecules remain in the well of CHEF gels, it is likely that the observation indicates that *GAL-POL1* strains have increased levels of DNA replication and/or recombination intermediates. Using two-dimensional gel electrophoresis, Zou and Rothstein [30] showed that certain mutants of DNA polymerases alpha and delta resulted in increased levels of an rDNA structure (termed “xDNA”) that had the properties expected for a recombination intermediate.

### Low Pol1p Dramatically Increases Meiotic rDNA Recombination

Meiotic recombination between rDNA clusters on homologous chromosomes is greatly suppressed. Although the rDNA is about 10% of the genome and the yeast genome has a genetic length of about 4200 cM, the rDNA cluster is only 2.5 cM in length [8],[20]. Unequal sister chromatid meiotic recombination is less suppressed, with loss of an internal marker occurring in up to 10% of tetrads [31]. To evaluate the effect of Pol1p levels on meiotic rDNA recombination, we sporulated the wild-type and *GAL-POL1* strains on plates containing either high or low galactose. Tetrads were dissected and scored for parental ditype (PD), non-parental ditype (NPD) and tetratype (T) for three intervals: *HPH*-*TRP1*, *TRP1*-*URA3*, and *HPH*-*URA3*. The genetic distances between markers were calculated by standard procedures [32] and are shown in Table 2. As expected, recombination in the rDNA in wild-type cells was extremely low, 1 cM for the entire cluster (*HPH-URA3* interval). Since the difference in recombination rates between the wild-type cells sporulated in high and low levels of galactose was not significant, the data were combined.

**Table 2:** Meiotic segregation patterns in wild-type and *GAL-POL1* strains.

Interval	Strain	PD	T	NPD	cM	NPD exp*	p†	Int‡
*HPH-TRP1*	wild-type§	343	3	0	0.4	0	nd	nd
	*GAL-POL1*, High Gal	201	17	1	5 (4)	0	nd	nd
	*GAL-POL1*, Low Gal	94	32	8	30 (12)	2.3	<.0001	−2.5
*TRP1-URA3*	wild-type§	342	4	0	1	0	nd	nd
	*GAL-POL1*, High Gal	207	10	2	5 (2)	0	nd	nd
	*GAL-POL1*, Low Gal	112	20	2	12 (8)	1	0.04	−2.3
*HPH-URA3*	wild-type§	339	7	0	1	0	nd	nd
	*GAL-POL1*, High Gal	190	25	4	11 (7)	1	<.0001	−5.7
	*GAL-POL1*, Low Gal	85	44	5	28 (19)	2.9	0.17	−0.72
*LYS2-TYR1*	wild-type§	163	250	3	32	22	<.0001	0.87
	*GAL-POL1*, High Gal	74	160	4	39	17	<.0001	0.77
	*GAL-POL1*, Low Gal	63	149	3	41	16	<.0001	0.82

Tetrads were examined from AMC45 (wild-type) and AMC20 (*GAL-POL1*). Cells from the two strains were sporulated under identical conditions on plates containing either high or low levels of galactose. CentiMorgan distances in parentheses for the *HPH*–*TRP1* and *TRP1*–*URA3* intervals are calculated without NPDs [32]; in the *HPH*–*URA3* interval, distances in parentheses are calculated using only those NPDs that are the result of a 4-strand double crossovers around *TRP1* (see text for details). Spore viability was 93% (wild-type, high galactose), 82% (*GAL-POL1*, high galactose), 90% (wild-type, low galactose), and 62% (*GAL-POL1*, low galactose). Sporulation efficiency was 17% (wild-type, high galactose), 19% (*GAL-POL1*, high galactose), 18% (wild-type, low galactose), and 17% (*GAL-POL1*, low galactose). PD, parental ditype; T, tetratype; NPD, nonparental ditype; cM, centiMorgans; nd, not determined because the expected value was 0.

*: Expected NPD tetrads if there is no interference, calculated using Stahl Lab Online Tools “A Better Way” (http://molbio.uoregon.edu/~fstahl/ and [35]).

**†:** Chi-square test, one degree of freedom.

**‡:** Degree of interference, calculated as 1 − (NPD_obs_/NPD_exp_).

**§:** Since there was no significant difference in the proportion of tetrad classes in cells sporulated on high and low galactose media for the wild-type cells, these data were combined.

In contrast, the genetic distance between *HPH* and *URA3* was increased to 28 cM in cells with low Pol1p (*p*<.0001). In the equation used to calculate map distances, in two-point crosses, NPD events are assumed to reflect four-strand double meiotic crossovers between markers [32]. For a two-point cross, however, an NPD event could also be a consequence of a mitotic crossover prior to meiosis. In general, the frequency of mitotic crossovers was lower than the observed frequency of meiotic NPD events, suggesting that at least some of the NPD tetrads reflect double meiotic crossovers. For example, in the strain with low levels of alpha DNA polymerase for the *HPH-TRP1* interval, we observed a rate of mitotic crossovers of 1% (Table 1), whereas the rate of NPDs for the same interval was 6% (Table 2). In addition, as will be discussed further below, in analyzing the *HPH-URA3* interval, we detected NPD tetrads that had one crossover in the *HPH-TRP1* interval and a second crossover in the *TRP1-URA3* interval, demonstrating that some NPD tetrads reflect meiotic exchanges. Nonetheless, we also calculated map distances for the three intervals excluding all of the NPD tetrads that could represent mitotic crossovers (values shown in parentheses in Table 2). Even with this conservative assumption, the genetic distance in the rDNA cluster is more than 10-fold elevated in the strain with low levels of alpha polymerase compared to the wild-type (*p*<.0001).

The genetic distances in the rDNA in cells with low Pol1p were not additive since the *HPH-TRP1* distance is 30 cM, the *TRP1-URA3* is 12 cM, and the *HPH-URA3* distance is only 28 cM. There are two likely interpretations of this non-additivity. First, as described above, some of the NPD tetrads used in calculated the *HPH-TRP1* and *TRP1-URA3* distances may reflect mitotic crossover events. Second, since the equation used to calculate map distance [32] is based on the assumption that the interval examined has two or fewer crossovers, map distances for intervals that have more than two crossovers are underestimated. We observed four tetrads from low Pol1p cells that had marker segregation patterns consistent with triple crossovers surrounding *TRP1*; no such tetrads were found in wild-type cells or in *GAL-POL1* cells sporulated on high galactose.

We also noted a significant increase in the *HPH-URA3* distance in *GAL-POL1* cells sporulated on high galactose relative to the wild-type strain, 11 and 1 cM, respectively (*p*<.0001). Pol1p is overexpressed about three-fold relative to wild-type under these conditions [26]. It is possible that the overexpression of this single unit of DNA polymerase alpha complex perturbs its assembly. We previously observed that overexpression of Pol1p resulted in elevated levels of chromosome rearrangements and chromosome loss [26].

We detected an elevation of the frequency of tetrads with one Trp^+^ and three Trp^−^ spores (instead of the expected 2∶2 marker segregation) in the *GAL-POL1* strain. Loss of the *TRP1* insertion can occur by unequal crossing-over between sister chromatids, intra-chromatid recombination, and gene conversion (either between homologues, or unequally between sister chromatids). Of the 280 four-spore tetrads from *GAL-POL1* cells sporulated on high galactose, 20 had one Trp^+^ to three Trp^−^ spores (7%). In *GAL-POL1* cells sporulated on low galactose, this level was 25% (51 out of 204 tetrads). We observed only one tetrad that had three Trp^+^ to one Trp^−^ spore, and this tetrad was from *GAL-POL1* cells sporulated on low galactose. This bias toward *TRP1* marker loss indicates that the majority of these events are intrachromatid events (for example, unequal crossovers between sisters), rather than “classic” gene conversion events. We did not observe *TRP1* marker loss in any tetrads from wild-type cells (total of 362 four-spore tetrads). This result differs from an earlier report in which an internal rDNA marker was lost in ∼10% of wild-type tetrads [31]. This variance may be due to differences in strain background or in the location of the inserted marker.

To clarify whether reduced Pol1p resulted in elevated meiotic crossovers in non-rDNA regions of the genome, we also investigated meiotic crossovers in a non-rDNA interval on chromosome II, between *LYS2* and *TYR1*. The *LYS2-TYR1* genetic distance in our wild-type strain is 32 cM, in agreement with the 35 cM average distance between these loci reported in the Saccharomyces Genome Database. In cells with low Pol1p, the distance between these markers was 41 cM (Table 2). Although this increase relative to wild-type was statistically significant (*p* = 0.02 by a Chi-square test), it is far less dramatic than that observed within the rDNA.

### Cells with Reduced Pol1p Have No Crossover Interference in the rDNA

In *S. cerevisiae*, as in most other eukaryotes, crossovers in one region reduce the probability of a nearby crossover [33]. In organisms in which tetrad analysis is possible, interference can be calculated in two-point crosses by analyzing the relative frequencies of PD, NPD, and T tetrads ([34]; modifications introduced by Stahl [35]; also, http://molbio.uoregon.edu/~fstahl/). For most genetic intervals in *S. cerevisiae*, NPD tetrads (representing four-strand double crossovers) are significantly less frequent than expected on the basis of the number of T tetrads (representing single crossovers as well as certain types of double crossovers). We used two procedures to calculate the expected number of NPD tetrads. First, using our observed numbers of PD, T, and NPD tetrads, we calculated the expected number of NPD tetrads by a direct application of the equation described in the Stahl Web site (NPD exp in Table 2). We then used a chi-square analysis to compare the observed and expected numbers of tetrads in all three classes, converting the chi square value to a *p* value (*p* in Table 2). We also calculated the degree of interference as 1−(NPD_observed_/NPD_expected_). For all three intervals in the strain with low levels of alpha DNA polymerase, interference was negative, suggesting that a crossover in one region of the rDNA increases the probability of a second crossover in the rDNA. This effect is specific to the rDNA, as a non-rDNA *LYS2-TYR1* interval on chromosome II has significant crossover interference in both the wild-type and *GAL-POL1* strains (Table 2).

Because (as discussed above), NPD tetrads in two-point crosses can reflect a mitotic exchange rather than two meiotic crossovers, we also examined interference in a more traditional way. From Table 2, we calculated that the frequency of tetratype tetrads (mostly representing single crossovers) in the *HPH-TRP1* interval in the *GAL-POL1* strain with low levels of DNA polymerase is about 0.25 (32 of 126 tetrads, excluding NPD tetrads from the total). The frequency of tetratype tetrads in the *TRP1-URA3* interval is 0.15. Thus, the expected frequency of tetrads with crossovers in both intervals (assuming no interference) is 0.04. In a sample of 134 tetrads examined in the *GAL-POL1* strain, we expect five DCOs in the *HPH-URA3* interval. We observed ten (Table S2). This calculation confirms that the crossovers observed in the rDNA in the *GAL-POL1* strain with low levels of alpha polymerase have no interference or negative interference.

### Increased Meiotic rDNA Recombination in Strains with Low Alpha DNA Polymerase Is Independent of the Fob1p-Dependent Replication Block

If the increased meiotic rDNA recombination in cells with low Pol1p is initiated from DSBs at the RFB site, we would expect the *fob1* mutation to reduce this recombination. Instead, we observed the opposite: the rate of recombination in cells that lack Fob1p and that have low Pol1p is significantly greater than observed in cells with only low Pol1p (Table 3), with a total *HPH-URA3* genetic distance of 50 cM (*p* = .04). We also found that *fob1* mutation alone significantly increased recombination relative to that observed in the wild-type strain (*p* = .0006). This finding is unexpected because, in mitosis, the *fob1* deletion reduces the rate of rDNA recombination [14], [17]–[19]. This difference in phenotype indicates that Fob1p has a unique role in meiosis separate from its role in mitosis.

**Table 3:** Meiotic segregation patterns in strains with the *fob1* mutation.

Interval	Strain	PD	T	NPD	cM	NPD exp	p	Int
*HPH-TRP1*	*POL1 fob1*	460	22	0	2	0	nd	nd
	*GAL-POL1 fob1*, High Gal	218	22	0	5	0	nd	nd
	*GAL-POL1 fob1*, Low Gal	125	36	3	17 (11)	1.4	0.15	−1.1
*TRP1-URA3*	*POL1 fob1*	463	19	0	2	0	nd	nd
	*GAL-POL1 fob1*, High Gal	203	35	2	10 (7)	0.8	0.17	−1.5
	*GAL-POL1 fob1*, Low Gal	113	43	8	28 (13)	2.8	.001	−1.9
*HPH-URA3*	*POL1 fob1*	445	37	0	4	0	nd	nd
	*GAL-POL1 fob1*, High Gal	192	45	3	13 (11)	1.4	0.15	−1.1
	*GAL-POL1 fob1*, Low Gal	86	61	17	50 (30)	7.8	<.0001	−1.2

Tetrads were examined from AMC156 (*POL1 fob1*) and AMC160 (*GAL-POL1 fob1*) sporulated on plates containing either high or low galactose levels. Column headings, abbreviations, and methods of analysis are identical to those in Table 2.

The frequencies of tetrads segregating one Trp^+^ to three Trp^−^ spores (indicating unequal sister chromatid or intrachromatid recombination) were 0.23 (*fob1 GAL-POL1* strain in low galactose), 0.12 (*fob1 GAL-POL1* strain in high galactose), and 0.014 (*fob1* strain). We also examined interference in these strains and, in the *GAL-POL1 fob1* cells, the observed number of NPDs was usually equal to or more than the expected number, indicating a lack of crossover interference (Table 3). In addition, in the *GAL-POL1 fob1* strain sporulated in low galactose, the expected frequency of double crossovers calculated from the frequencies of single crossovers in the *HPH-TRP1* interval (0.22) and the *TRP1-URA3* interval (0.26) is 0.06. Since the expected number of DCO tetrads is 10 (0.06×164 tetrads) and observed number is 11, there is no detectable crossover interference. Summing the data from the *GAL-POL1* and *fob1 GAL-POL1* strains sporulated in high or low galactose, we found 7 two-strand DCOs, 14 three-strand DCOs, and 9 four-strand double crossovers (Table S2). These numbers are close to the ratio predicted (1∶2∶1) if there is no chromatid interference.

### Increased Spo11p-Mediated Cleavage and Decreased Sir2p Binding of rDNA in Meiotic Cells with Low Pol1p

Meiotic recombination is initiated in *S. cerevisiae* by Spo11p-dependent DSBs [29]. The number of Spo11p-dependent DSBs in the rDNA is low, as expected based on the genetic data [22]. The hyper-Rec phenotype associated with low DNA polymerase could reflect either Spo11p-independent DSBs (perhaps generated during the meiotic S-period) or increased Spo11p-dependent DSBs. To distinguish between these two possibilities, we sporulated our strains under low-galactose conditions and used chromatin immunoprecipitation to purify Spo11p-associated DNA, followed by quantitative real-time PCR analysis. Since the chromatin immunoprecipitation experiments were done without formaldehyde treatment of the chromatin, these experiments monitor the covalent attachment of Spo11p to target DNA, reflecting Spo11p catalyzed DSBs. In both *POL1* and *GAL-POL1* cells, Spo11p-catalyzed DSBs were at same level at *HIS4*, a previously identified hotspot for Spo11p-mediated DSBs [36], with no significant difference between these strains (*p* = .487). There is an approximately 4-fold increase in Spo11p-associated rDNA in cells with low Pol1p compared to wild-type cells (*p* = .0003) (Figure S2 A–C). Thus, low levels of Polp1 disrupt mechanisms required for suppression of Spo11p entry into the rDNA array, leading to increased Spo11p-catalyzed DSBs.

Loss of the histone deacetylase Sir2p results in elevated rates of unequal meiotic [23] and mitotic unequal crossing over in the rDNA, and increased levels of Spo11p cleavage in the rDNA in meiotic cells [22]. We used quantitative real-time PCR of immunoprecipitated meiotic DNA to measure Sir2p in the *GAL-POL1* strain sporulated in low levels of galactose. In logarithmically-growing cells, there are two sites of Sir2p binding in each rDNA unit, one near the RFB site, and the other at the 5′ end of the 35S transcript [37]. We found that there is a significant decrease in Sir2p bound near the RFB site in *GAL-POL1* meiotic cells as compared to wild-type meiotic cells (*p* = .022) (Figure S2D). We also investigated Sir2p binding in logarithmically-growing cells with low Pol1p; we did not find a significant decrease in the level of Sir2p (data not shown). Lastly, by chromatin immunoprecipitation, we looked for an alteration in the binding of the cohesin subunit Mcd1p in vegetative wild-type cells and in cells with low Pol1p. We found no significant difference (data not shown).

## Discussion

We show that reduced levels of DNA polymerase alpha result in elevated mitotic recombination and greatly elevated meiotic recombination within the yeast rDNA. Unlike most previous studies of rDNA recombination, we used markers within and flanking the rDNA, allowing us to quantitate both sister-chromatid and homologue recombination. As described below, we suggest that the hyper-Rec phenotypes resulting from low alpha DNA polymerase in mitosis and meiosis reflect two fundamentally different mechanisms.

### Mitotic rDNA Recombination

The rate of reciprocal mitotic crossovers (RCOs) between homologues in the 120 kb *CEN5–CAN1* interval of chromosome V is about 4×10^−5^ per cell division [38]. Assuming this rate is representative of mitotic recombination throughout the genome, we would expect the rate of RCOs in the rDNA, which is about ten times larger than the *CEN5–CAN1* interval, to be approximately 4×10^−4^ per cell division. Since we observe a rate of RCOs of about 3×10^−3^ per cell division in the wild-type strain, mitotic crossovers in the rDNA are not suppressed and, in fact, appear somewhat elevated relative to non-rDNA sequences. In cells with low Pol1p, rDNA recombination between both clusters on homologues and clusters on sister chromatids was increased about five-fold. To determine whether stalling of replication forks at the RFB site is responsible for the elevated rDNA recombination, we examined strains that lacked the RFB-binding Fob1p. Although loss of Fob1p reduces the hyper-Rec rDNA phenotype associated with *sgs1* and *dna2* helicase mutants [13], [39]–[41], the *fob1* mutation did not decrease mitotic recombination in our strains with low polymerase. We also directly compared the level of DSBs at the RFB in wild-type and low Pol1p strains, and found no difference.

In previous studies, an elevated level of unequal sister-strand mitotic recombination in the rDNA was observed in strains lacking Sir2p [14],[23]. It is unlikely that the hyper-Rec effect of low alpha polymerase in mitotic cells reflects a reduction in the level of Sir2p for several reasons. First, loss of Sir2p specifically elevates rDNA recombination [23] but, as discussed below, loss of alpha polymerase elevates recombination in other regions of the genome. Second, the hyper-Rec phenotype caused by the *sir2* mutation is dependent on Fob1p [14], unlike the hyper-Rec phenotype resulting from low DNA polymerase alpha. Third, Kobayashi *et al.* [14] showed that intragenic recombination within a single rDNA gene was not elevated in *sir2* strains, although unequal sister-strand recombination was elevated. These researchers also found a defect in the level of the cohesin subunit Mcd1p in *sir2* strains and suggested that the loss of sister-strand cohesion in *sir2* strains led to elevated levels of unequal sister-strand recombination without an elevated level of recombinogenic lesions. Finally, we failed to see any effect of low alpha polymerase on the level of Sir2p binding in the rDNA in mitotic cells by chromatin immunoprecipitation experiments.

Although the hyper-Rec phenotype in our experiments is not correlated with elevated DSBs at the RFB, we suggest that the hyper-Rec phenotype is likely to reflect elevated DNA lesions (perhaps distributed randomly) based on several arguments. First, we found an elevated rate of RCO in an interval of chromosome XII (*CEN12-HPH*, Table 1) that does not contain rDNA, although only a small number of events were detected. Second, strains with low levels of alpha polymerase are hyper-Rec in non-rDNA regions; mitotic recombination in the *CEN5-CAN1* interval is elevated about twenty-fold by low alpha DNA polymerase [26]. A general hyper-Rec phenotype is associated with mutations affecting many components of the DNA replication system (reviewed by [42]). Third, an elevated level of DSBs in strains with low alpha DNA polymerase was physically demonstrated at a fragile site on chromosome III [26]. Fourth, increased levels of Holliday junctions, presumably representing repair of DNA lesions, are observed in the rDNA of polymerase alpha mutants [30]. Fifth, in analyzing intact chromosomal DNA samples by CHEF gels, chromosome XII was often trapped in the wells, characteristic of the behavior of branched DNA molecules. Strains that lack Rrm3 also have elevated levels of rDNA recombination and chromosome XII molecules that are trapped in the gel wells [43],[44].

### Meiotic rDNA Recombination

Low levels of Pol1p very substantially increase meiotic recombination in the rDNA between homologues and sister-chromatids. We also observe a small, but significant, elevation of recombination in the *LYS2-TYR1* interval (Table 1). The much greater stimulation of recombination in the rDNA and the observed increase in Spo11p-mediated DSBs in the rDNA in strains with low levels of DNA polymerase argue that the stimulation is not primarily a consequence of DSBs associated with problems with DNA replication. The stimulation is also independent of Fob1p. In contrast to its effect in mitosis, loss of Fob1p results in increased rather than decreased meiotic recombination in the rDNA. Fob1p is involved in the recruitment of Sir2p to the rDNA [45]. Since we found that strains with low alpha DNA polymerase have somewhat reduced meiotic levels of Sir2p in the rDNA, two different methods of reducing the concentration of Sir2p in the nucleolus result in a hyper-Rec meiotic phenotype.

Based on our observations and those of others, a relatively simple model can be proposed. A reduction in the level of Sir2p in the rDNA results in a reduction in the level of the Pch2p. San-Segundo and Roeder [24] showed that Sir2p was required for the localization of Pch2p to the nucleolus and *pch2* mutants had elevated rates of meiotic recombination. These researchers also showed that Pch2p excludes Hop1p from the nucleolus. Since *hop1* strains have reduced levels of Spo11p-catalyzed DSBs [46], increased entry of Hop1p into the nucleolus would be expected to elevate Spo11p-induced DSBs.

There are several alternative explanations of our data. First, breaks in the rDNA of cells with low Pol1p during meiotic S-phase may be re-located outside the nucleolus for repair, and during this time of re-location, Spo11-induced DSBs could be formed [47]. Second, DNA lesions (for example, single-strand nicks) in the rDNA in cells with low polymerase may recruit the Mre11p/Rad50p/Xrs2p complex that subsequently associates with Spo11p [48], resulting in an increased level of Spo11p-catalyzed DSBs. Finally, we cannot rule out the possibility that some of the meiotic recombinogenic lesions are a consequence of DNA lesions resulting from low DNA polymerase during meiotic recombination that are independent of Spo11p.

In summary, we suggest that *fob1* mutations have different recombination phenotypes in mitosis (hypo-Rec) and meiosis (hyper-Rec) because of the different effects of Sir2p. In mitosis, the primary recombination-related role of Sir2p is to help maintain sister-chromatid cohesion and loss of Sir2p results in elevated unequal sister-strand recombination. In meiotic recombination, Sir2p acts to prevent recombination-stimulating proteins such as Hop1p and Spo11p from entering the nucleolus and, consequently, loss of Sir2p elevates meiotic recombination.

These explanations leave two important questions unanswered. First, why does a low level of alpha DNA polymerase reduce Sir2p binding in the rDNA of meiotic cells? Second, why does low alpha DNA polymerase reduce Sir2p binding in meiotic, but not mitotic cells? Although we cannot provide definitive answers to either of these questions, it is possible that the role of Pol1p in chromatin assembly is relevant. Pol1p interacts with Spt16p-Pob3p (components of the nucleosome reorganization complex) and Ctf4p, a protein involved in sister-chromatid cohesion [49]. Consequently, a severe reduction in the level of alpha DNA polymerase might affect the replication-associated assembly of DNA-interacting proteins, including Sir2p, within the rDNA. Although it is not clear why this effect would be observed in meiotic, but not mitotic cells, there are a substantial number of differences between the meiotic and mitotic S-phases including the length of the S-phase and the proteins required for S (reviewed by [50]).

Meiotic crossovers in the rDNA in strains with low levels of alpha DNA polymerase exhibit no chiasma interference. Low alpha polymerase does not result in a general loss of interference, since we still observed interference between *LYS2* and *TYR1*. Since it has been argued that crossovers that occur outside of the context of the synaptonemal complex show no interference (reviewed by [51]) and no distinct synaptonemal complex is present in the nucleolus [52], this result is not unexpected. More recent observations, however, suggest that crossover interference can occur in the absence of synaptonemal complex formation [53],[54]. Whatever the mechanism by which one crossover reduces the probability of a local second crossover, this mechanism does not operate for crossovers in the rDNA. One final important point is that we do not know whether low levels of alpha DNA polymerase alter interference in the rDNA. In wild-type strains, the frequency of single crossovers in the rDNA is so low (about 1% of the tetrads) that it is difficult to determine whether the double crossovers are less than expected. Finally, Hsueh *et al.* [55] recently reported that crossovers flanking the mating-type locus in *Cryptoccus* show negative interference.

In summary, we show that the rDNA array is destabilized by high levels of recombination under conditions of low levels of DNA polymerase alpha. The hyper-Rec phenotype is not due to increased breaks at the RFB, and cannot be rescued by deletion of *fob1*. The hyper-Rec phenotypes observed in mitosis and meiosis appear to reflect two different mechanisms: a general hyper-Rec phenotype in mitotic cells (possibly resulting from increased levels of DSBs throughout the genome) and a more specific hyper-Rec phenotype in meiotic cells (reflecting increased entry of Spo11p into the nucleolus). Our analysis shows that the coordination of replication and recombination is critical for the maintenance of rDNA stability both in mitosis and in meiosis.

## Materials and Methods

### Strain Constructions

The strains in this study were isogenic with MS71, a *LEU2* derivative of AMY125 (*α ade5-1 leu2-3 trp1-289 ura3-52 his7-2*) [56], except for changes introduced by transformation. The diploids used for most of our analysis (all having the markers shown in Figure 1) were: AMC45 (wild-type), AMC20 (*GAL-POL1*), AMC156 (*fob1*), and AMC160 (*GAL-POL1 fob1*). Strain constructions and complete genotypes for all strains are in Tables S3 and S4.

### Genetic Methods and Media

Methods of transformation, mating, and tetrad dissection were standard. Strains were grown at 30° and sporulated at 25°C. High-galactose media contained 0.05% galactose and low-galactose media contained 0.005% galactose, as well as 3% raffinose, plus the standard supplements of yeast extract and peptone; dextrose was omitted. Selective media and sporulation media were standard, except for the addition of high or low galactose, and the substitution of dextrose with raffinose [57].

### CHEF Analysis and Southern Analysis of DSBs at the RFB

Genomic DNA was extracted and treated with restriction enzymes in agarose plugs to avoid shearing; further descriptions of plug preparation and restriction-enzyme digestion are included in Text S1. For CHEF analysis, DNA was subjected to electrophoresis at 14°C in a 1.0% gel, 0.5× TBE buffer, using a BioRad CHEF Mapper XA, with switch times starting at 47 sec. and extending to 2 min. 49 sec. at 5 V/cm for 33 hr. For analysis of DSBs at the RFB, DNA was subjected to horizontal gel electrophoresis at room temperature in a 1.0% gel, 1× TBE buffer, for 16 hr at 1.3 V/cm. Gels were stained with ethidium bromide, photographed, and then used for Southern analysis.

To analyze the CHEF gels, we used an rDNA-specific probe prepared by treating the pY1rG12 rDNA plasmid [58] with *Eco*RI. For Southern analysis of DSBs at the RFB (rDNA probe #1), we performed PCR of genomic DNA with the primer pair: 5′ GTTAAGCACTCCATTATG 3′ and 5′ TAGTTAACAGGACATGCC 3′. Probes were labeled by random-prime labeling using Ready-To-Go DNA Labeling Beads (GE Healthcare). Southern hybridization and washing were standard. Membranes were exposed to a PhosphoImager screen for one to three days. Images were captured with a Typhoon imager (GE Healthcare) and quantification was performed using Quantity One analysis software (BioRad).

### Chromatin Immunoprecipitation

Immunoprecipitation and real-time PCR experiments were standard, and are described in Text S1.

### Statistical Analyses

Calculations of 95% confidence intervals and *p*-values using Chi Square and Fisher's Exact tests were done using VassarStats (http://faculty.vassar.edu/lowry/VassarStats.html). The number of NPDs expected in each interval was calculated using the Stahl Lab Online Tools “Better Way” calculator (http://www.molbio.uoregon.edu/~fstahl/ and also [35]). This calculation was used instead of the traditional NPD = 1/2[1−T−(1–3T/2)^2/3^] [34], because in the Papazian equation, the expected number of NPDs cannot be calculated when T exceeds 2/3. In the *LYS2-TYR1* interval, T in some strains exceeds 2/3. The “Better Way” calculator is better for all values of T and can be applied when T>2/3.

## Supporting Information

Figure S1 Under-representation of chromosome XII in a strain with low levels of DNA polymerase alpha. (A) Analysis of chromosome migration by CHEF gel separation of genomic DNA from early log-phase cultures of wild-type cells in YPD (lane 1) and *GAL-POL1* cells in YPR with low galactose (lane 2). (B) Southern blot of the gel shown in (A) with an rDNA-specific probe.
(0.45 MB TIF)

Figure S2 ChIP analysis of Sir2p- and Spo11p-associated DNA in a wild-type strain and a strain with low levels of Pol1p. (A) Location of primer sets within the rDNA used for real-time PCR analysis of chromatin immunoprecipitations; primers used to generate the PCR products are in Supp. Table 5. A single rDNA unit is shown; PCR products are a selection of those published by Huang and Moazed [5] and are indicated by black horizontal bars. (B) and (C) Spo11p-associated DNA immunoprecipitated from wild-type (*POL1*) and *GAL-POL1* strains sporulated in low galactose was quantified by real-time PCR. Spo11p binding at *HIS4* (a known hotspot for Spo11p binding) and at rDNA location 19 were quantified relative to Spo11p binding at the *CUP1* locus. Error bars represent the 95% confidence intervals. (D) Sir2p-associated DNA was immunoprecipitated from wild-type (*POL1*) and *GAL-POL1* strains sporulated in low galactose. The binding was quantified by real-time PCR, relative to binding at rDNA location 3-3. Error bars represent the 95% confidence intervals.
(0.33 MB TIF)

Table S1 Frequencies of sectored colonies of various phenotypes reflecting mitotic recombination in a *fob1* strain and in *fob1* strains with high and low levels of DNA polymerase alpha. Colonies of AMC156 (*fob1*) and AMC157 (*fob1 GAL-POL1*), grown on rich medium, were replica-plated to medium lacking uracil or tryptophan, or containing hygromycin. As in Table 1, the patterns of sectoring and other types of analysis (primarily tetrad analysis of the sectors) were used to classify the different types of recombination events. A full discussion of this classification is in the Table 1 legend.
(0.03 MB DOC)

Table S2 Numbers of two-, three-, and four-strand meiotic double crossovers (DCOs) in tetrads with a crossover in the *HPH-TRP1* and *TRP1-URA3* intervals.
(0.03 MB DOC)

Table S3 Haploid strain genotypes and constructions. *All strains were derived from MS71 (α *ade5-1 his7-2 ura3-52 trp1-289*) by transformation or crosses with isogenic strains. Only those markers that differ from the genotype of MS71 are shown. Some of our strains contain insertion of drug-resistant markers at genomic locations that are not within genes. For such markers, we indicate the chromosome containing the insertion and the SGD coordinate at the position of the insertion. For example, the marker *XII451250::HPH* represents an insertion of the hygromycin-resistance gene on chromosome XII next to base 451250. **Strains constructed by transformation were made using PCR fragments to the targeted location. The template for PCR amplification is indicated. Primer sequences used in strain construction are shown with upper case letters corresponding to the targeted genomic regions and lower case letters corresponding to the selectable marker on the plasmid.
(0.08 MB DOC)

Table S4 Diploid strain genotypes and constructions. *All strains were derived by crosses of haploids that are isogenic with MS71 (described in Supp. Table 4). Only those markers that differ from the genotype of MS71 are shown. An illustration of the nomenclature for a diploid heterozygous for an insertion of a drug-resistant marker is: *XII451250::HPH/ XII451250*. In this strain, one chromosome had an insertion of the *HPH* gene on chromosome XII at base 451250 (SGD coordinates) and the other chromosome did not.
(0.03 MB DOC)

Table S5 Primers for real-time PCR.
(0.04 MB DOC)

Text S1 Low levels of DNA polymerase alpha induce mitotic and meiotic instability in the ribosomal DNA gene cluster of *Saccharomyces cerevisiae*.
(0.05 MB DOC)
