# Supplementary figures and images for: Low Levels of DNA Polymerase Alpha Induce Mitotic and Meiotic Instability in the Ribosomal DNA Gene Cluster of Saccharomyces cerevisiae
Source: PLoS Genet. 2008 Jun 27;4(6):e1000105. doi: 10.1371/journal.pgen.1000105 (PMC2430618; doi:10.1371/journal.pgen.1000105)

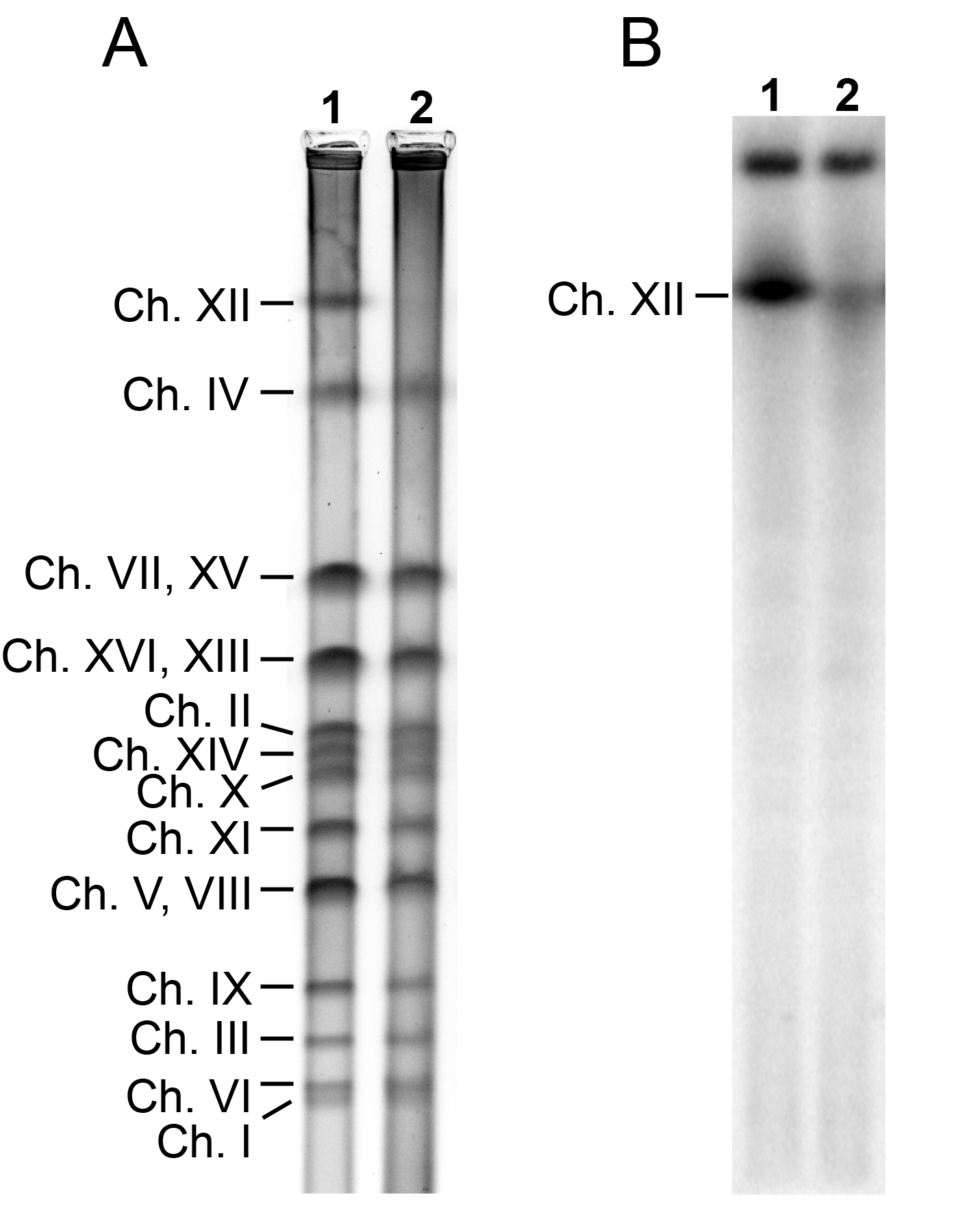

Supplement: Figure S1 — Under-representation of chromosome XII in a strain with low levels of DNA polymerase alpha. (A) Analysis of chromosome migration by CHEF gel separation of genomic DNA from early log-phase cultures of wild-type cells in YPD (lane 1) and GAL-POL1 cells in YPR with low galactose (lane 2). (B) Southern blot of the gel shown in (A) with an rDNA-specific probe. (0.45 MB TIF) [file pgen.1000105.s001.tif]

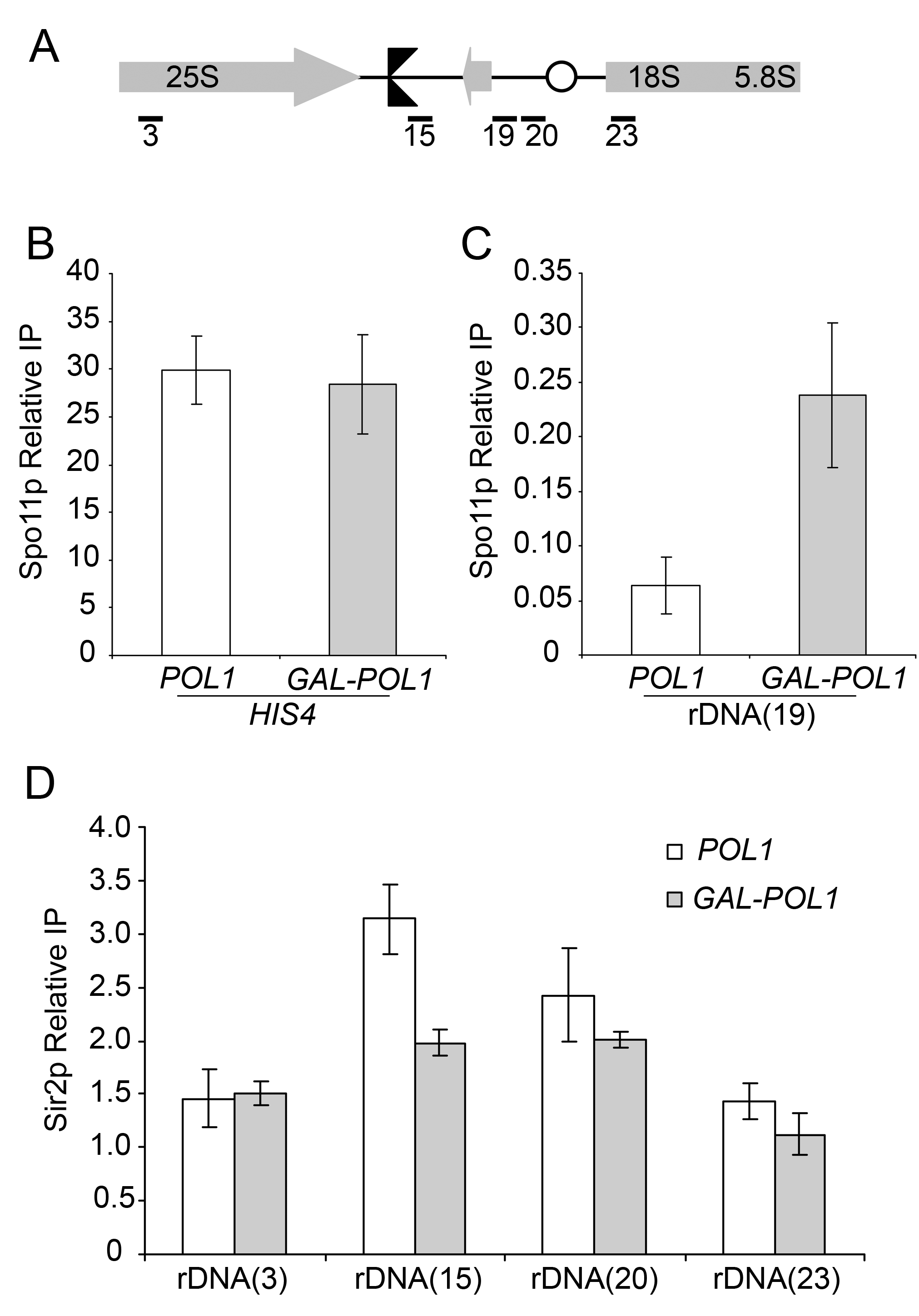

Supplement: Figure S2 — ChIP analysis of Sir2p- and Spo11p-associated DNA in a wild-type strain and a strain with low levels of Pol1p. (A) Location of primer sets within the rDNA used for real-time PCR analysis of chromatin immunoprecipitations; primers used to generate the PCR products are in Supp. Table 5. A single rDNA unit is shown; PCR products are a selection of those published by Huang and Moazed [5] and are indicated by black horizontal bars. (B) and (C) Spo11p-associated DNA immunoprecipitated from wild-type (POL1) and GAL-POL1 strains sporulated in low galactose was quantified by real-time PCR. Spo11p binding at HIS4 (a known hotspot for Spo11p binding) and at rDNA location 19 were quantified relative to Spo11p binding at the CUP1 locus. Error bars represent the 95% confidence intervals. (D) Sir2p-associated DNA was immunoprecipitated from wild-type (POL1) and GAL-POL1 strains sporulated in low galactose. The binding was quantified by real-time PCR, relative to binding at rDNA location 3-3. Error bars represent the 95% confidence intervals. (0.33 MB TIF) [file pgen.1000105.s002.tif]
