# Supplementary material for: Low Levels of DNA Polymerase Alpha Induce Mitotic and Meiotic Instability in the Ribosomal DNA Gene Cluster of Saccharomyces cerevisiae
Source: PLoS Genet. 2008 Jun 27;4(6):e1000105. doi: 10.1371/journal.pgen.1000105 (PMC2430618; doi:10.1371/journal.pgen.1000105)
Supplement: Table S1 — Frequencies of sectored colonies of various phenotypes reflecting mitotic recombination in a fob1 strain and in fob1 strains with high and low levels of DNA polymerase alpha. Colonies of AMC156 (fob1) and AMC157 (fob1 GAL-POL1), grown on rich medium, were replica-plated to medium lacking uracil or tryptophan, or containing hygromycin. As in Table 1, the patterns of sectoring and other types of analysis (primarily tetrad analysis of the sectors) were used to classify the different types of recombination events. A full discussion of this classification is in the Table 1 legend. (0.03 MB DOC) [file pgen.1000105.s003.doc]

|  |  | **rDNA recombination between homologues** | | | | | |  |
| --- | --- | --- | --- | --- | --- | --- | --- | --- |
| Genotype, growth condition, and number of colonies | *CEN12-HPH*  RCO | *HPH-TRP1*  RCO | *TRP1*-*URA3*  RCO | BIR initiated  on *URA3*  chromatid | Gene  conversion | BIR initiated  on *HPH*  chromatid | Total | Sister  chromatid  recombination |
| ***fob1***  n = 3794 colonies | 1 (2 x 10^-4^) | 0 | 0 | 1 (2 x 10^-4^) | 0 | 0 | **2 x 10^-4^** | 3 (8 x 10^-4^) |
| ***fob1 GAL-POL1***  **High Gal**  n = 2252 colonies | 1 (9 x 10^-4^) | 1 (9 x 10^-4^) | 0 | 0 | 0 | 1 (4 x 10^-4^) | **1.3 x 10^-3^** | 2 (9 x 10^-4^) |
| ***fob1 GAL-POL1***  **Low Gal**  n = 2072 colonies | 0 | 12 (10^-2^) | 12 (10^-2^) | 1 (5 x 10^-4^) | 1 (5 x 10^-4^) | 0 | **2 x 10^-2^** | 20 (10^-2^) |
