# Supplementary material for: Low Levels of DNA Polymerase Alpha Induce Mitotic and Meiotic Instability in the Ribosomal DNA Gene Cluster of Saccharomyces cerevisiae
Source: PLoS Genet. 2008 Jun 27;4(6):e1000105. doi: 10.1371/journal.pgen.1000105 (PMC2430618; doi:10.1371/journal.pgen.1000105)
Supplement: Table S2 — Numbers of two-, three-, and four-strand meiotic double crossovers (DCOs) in tetrads with a crossover in the HPH-TRP1 and TRP1-URA3 intervals. (0.03 MB DOC) [file pgen.1000105.s004.doc]

| **Strain, growth condition, number of tetrads** | **Two-strand DCO** | **Three-strand DCO** | **Four-strand DCO** | **Total DCO** |
| --- | --- | --- | --- | --- |
| *GAL-POL1*, High Gal  n = 219 tetrads | 0 | 1 | 1 | 2 |
| *GAL-POL1*, Low Gal  n = 134 tetrads | 2 | 7 | 1 | 10 |
| *fob1 GAL-POL1*, High Gal  n = 240 tetrads | 4 | 2 | 1 | 7 |
| *fob1 GAL-POL1*, Low Gal  n = 164 tetrads | 1 | 4 | 6 | 11 |
