# Supplementary material for: Low Levels of DNA Polymerase Alpha Induce Mitotic and Meiotic Instability in the Ribosomal DNA Gene Cluster of Saccharomyces cerevisiae
Source: PLoS Genet. 2008 Jun 27;4(6):e1000105. doi: 10.1371/journal.pgen.1000105 (PMC2430618; doi:10.1371/journal.pgen.1000105)
Supplement: Table S3 — Haploid strain genotypes and constructions. *All strains were derived from MS71 (α ade5-1 his7-2 ura3-52 trp1-289) by transformation or crosses with isogenic strains. Only those markers that differ from the genotype of MS71 are shown. Some of our strains contain insertion of drug-resistant markers at genomic locations that are not within genes. For such markers, we indicate the chromosome containing the insertion and the SGD coordinate at the position of the insertion. For example, the marker XII451250::HPH represents an insertion of the hygromycin-resistance gene on chromosome XII next to base 451250. **Strains constructed by transformation were made using PCR fragments to the targeted location. The template for PCR amplification is indicated. Primer sequences used in strain construction are shown with upper case letters corresponding to the targeted genomic regions and lower case letters corresponding to the selectable marker on the plasmid. (0.08 MB DOC) [file pgen.1000105.s005.doc]

| **Strain Name** | Relevant Genotype* | Reference or Construction Details** |
| --- | --- | --- |
| EAS18 | *a* | [8] |
| NPD1 | *KANMX-GAL1-POL1* | [8] |
| NPD44 | *a KANMX-GAL1-POL1 can1 his1::HPH HIS7* | [8] |
| AMC2 | *KANMX-GAL1-POL1* | Spore colony from NPD1 x NPD44 cross |
| AMC3 | *a KANMX-GAL1-POL1* | Spore colony from NPD1 x NPD44 cross |
| AMC10 | *a KANMX-GAL1-POL1 XII451250::HPH* | Transformation of AMC3 with *HPH* targeted centromere-proximal to rDNA array; pAG32 template [9]; primers AMC001(5'ACTGGGAACAATCTT  GCGGCACACCGCTTGAACGAAACGTTCGTAAcgtacgctgcaggtcgac) and AMC002 (5'AATATGAAACAATGATAATAAATATTAAGATTATTAAATTATATATatcgatgaattcgagctcg) |
| AMC17 | *a KANMX-GAL1-POL1 XII451250::HPH XII460158::TRP1* | Transformation of AMC10 with *TRP1* targeted within rDNA array; AS13 genomic DNA template [10]; primers AMC026 (5’ GCTTCTTATT  CCTTCCCGCTTTCCTGCACTAACATTTTGCCGCATTACACtgaatgaacgtatacgcg and AMC027 (5’CGACGCGGCGACGCGGTATGCGGAGTTGTAAGATGTAC  TACGATCATATAgtgaaggagcatgttcgg) |
| AMC18 | *KANMX-GAL1-POL1 XII490694::K.l.URA3* | Transformation of AMC2 with *K. lactis URA3* targeted cetromere-distal to rDNA array; pCORE template [11]; primers AMC023 (5’TATATATGGATTGAATATTAGAGTTA  CCCTACGTTCAATTTCTAGATTCTgttacctcactcattagg) and AMC024(5’GTAAAATAGGTAGATAGGACAGATGCAGAACTCATCAGGGGTAACACCATgttgaagtgagtgttgcac) |
| AMC31 | *a KANMX-GAL1-POL1 XII490694::K.l.URA3* | Spore colony from AMC17 x AMC18 cross |
| AMC32 | *XII490694::K.l.URA3* | Spore colony from AMC31 x MS71 cross |
| AMC34 | *a XII451250::HPH XII460158::TRP1* | Spore colony from AMC17 x MS71 cross |
| AMC88 | *XII490694::K.l.URA3 fob1::NAT* | Transformation of AMC32 with *fob1::NAT*; pAG25 template [9]; primers AMC065 (5'GGAGAACAATTTAACGATTGTGTGAGTGTGAATTTGTGCT  GAGGATAACAcgtacgctgcaggtcgac) and AMC066 (5'AACCGCGTACATTAAATACA  GGGTCATATACAGGAAGAGCTTTCAACACCatcgatgaattcgagctcg) |
| AMC89 | *a XII451250::HPH XII460158::TRP1 fob1::NAT* | Transformation of AMC34 with *fob1::NAT*; pAG25 template [9]; primers AMC065 and AMC066, as above |
| AMC94 | *KANMX-GAL1-POL1 XII490694::K.l.URA3 fob1::NAT* | Spore colony from AMC18 x AMC89 cross |
| AMC103 | *a KANMX-GAL1-POL1 XII451250::HPH XII460158::TRP1 fob1::NAT* | Transformation of AMC17 with *fob1::NAT*; pAG25 template [9]; primers AMC065 and AMC066, as above |
| AMC132 | *a XII451250::HPH XII460158::TRP1 fob1::NAT* | Transformation of AMC34 with *fob1::NAT*; pAG25 template [9]; primers AMC065 and AMC066, as above |
| AMC136 | *KANMX-GAL1-POL1 XII490694::K.l.URA3 lys2* | Spore colony from AMC18 x AMC142 cross |
| AMC142 | *a XII451250::HPH XII460158::TRP1 lys2* | Amino-adipate resistant colony of AMC34 (*lys2* mutation confirmed by complementation testing) |
| AMC144 | XII490694::K.l.URA3 tyr1::NAT | Transformation of AMC32 with *tyr1::NAT*; pAG25 template [9]; primers AMC090 (5’CATCTCGTTGCCAATGAGATTAGCCTCTGGAAGCATTGGC  TCAAAGAACTcgtacgctgcaggtcgac) and AMC091(5’GAATACCGTAGCACTT  GAAGGAAAGAGGACAGCATATCCACTTGATAAACatcgatgaattcgagctcg) |
| AMC148 | *a KANMX-GAL1-POL1 XII451250::HPH XII460158::TRP1 tyr1::NAT* | Spore colony from AMC17 x AMC144 cross |
| AMC162 | *MCD1-ZZ::K.l.URA3* | Transformation of MS71 with PCR product containing two tandem epitopes of protein A of *S. aureus* (ZZ epitope) and *K. lactis URA3*, targeted to tag the 3’ end of *MCD1* with the ZZ epitope; pBS1365 template [12]; primers MCD1URA3F(5’GGAAATATTAAAATAGACGCCAAACCTGCACTATTTGAAAGGTTTATCAATGCTTAaagctggagctcaaaac)  and MCD1URA3R(5’GTCTTTGATCTATATATGCATCAGCTTATTGGGTCCACCAAGAAATCCCCTCGGCGTacgactcactataggg) |
| AMC164 | *a MCD1-ZZ::K.l.URA3* | Transformation of EAS18 with PCR product containing two tandem epitopes of protein A of *S. aureus* (ZZ epitope) and *K. lactis URA3*, targeted to tag the 3’ end of *MCD1* with the ZZ epitope; pBS1365 template [12]; primers MCD1URA3F and MCD1URA3R, as above |
| AMC168 | *KANMX-GAL1-POL1 MCD1-ZZ::K.l.URA3* | Spore colony from AMC2 x AMC164 cross |
| AMC170 | *a KANMX-GAL1-POL1 MCD1-ZZ::K.l.URA3* | Spore colony from AMC3 x AMC162 cross |
| AMC180 | *sae2::HPH* | Transformation of MS71 with *sae2::HPH*; pAG32 template [9]; primers AMC113 (5’AATGTGTATCTAAAGTCAAGCTTATC CATTCTCAAGGAGCTCAGTCTCGAcgtacgctgcaggtcgac) and AMC114 (5’CTTT CTTCTGATGATTTCCTGGGATTTCTTTTTGTCCTCGTTCCCTTCCTatcgatgaattcgagctc) |
| AMC185 | *sae2::HPH SPO11-ZZ::K.l.URA3* | Transformation of AMC180 with PCR product containing two tandem epitopes of protein A of *S. aureus* (ZZ epitope) and *K. lactis URA3*, targeted to tag the 3’ end of *SPO11* with the ZZ epitope; pBS1365 template [12]; primers SPO11URA3F(5’AGAAAGCTGAAATGAACGAGATTGATGCCAGAATTTTTGAATACAAAaagctggagctcaaaac) and SPO11URA3R(5’TTTCAATTCTTGAAAAA  CATTTTTTATAAAGCAACAGCTCCCATTCTTATTacgactcactataggg) |
| AMC190 | *KANMX-GAL1-POL1 sae2::HPH*  *SPO11-ZZ::K.l.URA3* | Spore colony from AMC3 x AMC185 cross |
| AMC191 | *a KANMX-GAL1-POL1 sae2::HPH*  *SPO11-ZZ::K.l.URA3* | Spore colony from AMC3 x AMC185 cross |
| AMC192 | *a sae2::HPH SPO11-ZZ::K.l.URA3* | Spore colony from AMC3 x AMC185 cross |
