# Supplementary material for: Low Levels of DNA Polymerase Alpha Induce Mitotic and Meiotic Instability in the Ribosomal DNA Gene Cluster of Saccharomyces cerevisiae
Source: PLoS Genet. 2008 Jun 27;4(6):e1000105. doi: 10.1371/journal.pgen.1000105 (PMC2430618; doi:10.1371/journal.pgen.1000105)
Supplement: Table S4 — Diploid strain genotypes and constructions. *All strains were derived by crosses of haploids that are isogenic with MS71 (described in Supp. Table 4). Only those markers that differ from the genotype of MS71 are shown. An illustration of the nomenclature for a diploid heterozygous for an insertion of a drug-resistant marker is: XII451250::HPH/ XII451250. In this strain, one chromosome had an insertion of the HPH gene on chromosome XII at base 451250 (SGD coordinates) and the other chromosome did not. (0.03 MB DOC) [file pgen.1000105.s006.doc]

| **Strain Name** | **Cross** | **Relevant Genotype*** |
| --- | --- | --- |
| AMC20 | AMC17 x AMC18 | *a/α KANMX-GAL1-POL1/KANMX-GAL1-POL1 XII451250::HPH/ XII451250 XII460158::TRP1/ XII460158 XII490694/XII490694::K.l.URA3* |
| AMC45 | AMC32 x AMC34 | *a/α XII451250::HPH/ XII451250 XII460158::TRP1/ XII460158 XII490694/XII490694::K.l.URA3* |
| AMC151 | AMC142 x AMC144 | *a/α  XII451250::HPH/ XII451250 XII460158::TRP1/ XII460158 XII490694/XII490694::K.l.URA3 lys2/ LYS2 TYR1/tyr1::NAT* |
| AMC152 | AMC136 x AMC148 | *a/α KANMX-GAL1-POL1/KANMX-GAL1-POL1 XII451250::HPH/ XII451250 XII460158::TRP1/ XII460158 XII490694/XII490694::K.l.URA3 lys2/ LYS2 TYR1/tyr1::NAT* |
| AMC156 | AMC88 x AMC132 | *a/α fob1::NAT/fob1::NAT XII451250::HPH/ XII451250 XII460158::TRP1/ XII460158 XII490694/XII490694::K.l.URA3* |
| AMC160 | AMC94 x AMC103 | *a/α fob1::NAT/fob1::NAT KANMX-GAL1-POL1/KANMX-GAL1-POL1 XII451250::HPH/ XII451250 XII460158::TRP1/ XII460158 XII490694/XII490694::K.l.URA3* |
| AMC166 | AMC162 x AMC164 | *a/α MCD1-ZZ::K.l.URA3/MCD1-ZZ::K.l.URA3* |
| AMC172 | AMC168 x AMC170 | *a/α KANMX-GAL1-POL1/KANMX-GAL1-POL1 MCD1-ZZ::K.l.URA3/MCD1-ZZ::K.l.URA3* |
| AMC193 | AMC185 x AMC192 | *a/α sae2::HPH/sae2::HPH SPO11-ZZ::K.l.URA3/SPO11-ZZ::K.l.URA3* |
| AMC194 | AMC190 x AMC191 | *a/α sae2::HPH/sae2::HPH KANMX-GAL1-POL1/KANMX-GAL1-POL1 SPO11-ZZ::K.l.URA3/SPO11-ZZ::K.l.URA3* |
