# Supplementary material for: Low Levels of DNA Polymerase Alpha Induce Mitotic and Meiotic Instability in the Ribosomal DNA Gene Cluster of Saccharomyces cerevisiae
Source: PLoS Genet. 2008 Jun 27;4(6):e1000105. doi: 10.1371/journal.pgen.1000105 (PMC2430618; doi:10.1371/journal.pgen.1000105)
Supplement: Table S5 — Primers for real-time PCR. (0.04 MB DOC) [file pgen.1000105.s007.doc]

| **Locus** | **Primer Sequences** |
| --- | --- |
| *CARC1* [7] | AMC147 (f)III-101346 (5’ GATCAATAGGGGTTCCATCG) and  AMC148 (r)III-101497 (5’ CGACGTAAGAGGTGGTTTGG) |
| *HIS4* | AMC135 (f)III-68025 (5’ CACAACAACACGCTCCTTTG) and    AMC136 (r)III-68271 (5’ CGTTTCACTTGTTGGTCAGG) |
| *CUP1* | CUP1-F (5’ TGAAGG TCATGAGTGCCAAT) and  CUP1-R (5’ TTCGTTTCATTTCCCAGAGCA) |
| rDNA primer set 3 | RDN3 U (5’CCATTATGCCAGCATCCTTG) and  RDN3 L (5’ GTAGCTTGCCTCGGTAAGTA) |
| rDNA primer set 3-3 | RDN3-3 U (5’ CGCCGTTACTAAGGCAATCC ) and  RDN3-3 L (5’ GAGCGTCTAGGCGAACAATG) |
| rDNA primer set 15 [5] | RDN-F15 (5’ AGGGCTTTCACAAAGCTTCC) and  RDN-R15 (5’ TCCCCACTGTTCACTGTTCA) |
| rDNA primer set 19 [5] | RDN-F19 (5’ GAGGTGTTATGGGTGGAGGA) and  RDN-R19 (5’ GCCACCATCCATTTGTCTTT) |
| rDNA primer set 20 [5] | RDN-F20 (5’ TGCAAAAGACAAATGGATGG) and  RDN-R20 (5’ GCACCTTTTCCTCTGTCCAC) |
| rDNA primer set 23 [5] | RDN-F23 (5’ GGGAGGTACTTCATGCGAAA) and  RDN-R23 (5’ AAGATGCCCACGATGAGACT) |
