# Supplementary material for: Low Levels of DNA Polymerase Alpha Induce Mitotic and Meiotic Instability in the Ribosomal DNA Gene Cluster of Saccharomyces cerevisiae
Source: PLoS Genet. 2008 Jun 27;4(6):e1000105. doi: 10.1371/journal.pgen.1000105 (PMC2430618; doi:10.1371/journal.pgen.1000105)
Supplement: Text S1 — Low levels of DNA polymerase alpha induce mitotic and meiotic instability in the ribosomal DNA gene cluster of Saccharomyces cerevisiae. (0.05 MB DOC) [file pgen.1000105.s008.doc]

**Text S1**

**Low levels of DNA polymerase alpha induce**

**mitotic and meiotic instability in the ribosomal DNA gene**

**cluster of *Saccharomyces cerevisiae***

Anne M. Casper*, Piotr A. Mieczkowski, Malgorzata Gawel and Thomas D. Petes

Department of Molecular Genetics and Microbiology, Box 3054, Duke University Medical Center, Durham, NC 27710

**Supporting Materials and Methods:**

**Strain constructions**

All strains used in this study were derived from MS71, a *LEU2* derivative of AMY125 [1]. The genotype of AMY125 is *α ade5-1 leu2-3 trp1-289 ura3-52 his7-2*. Haploid and diploid strains are described in Supp. Tables 3 and 4, respectively.

**Detailed analysis of Trp^+^/Trp^-^ sectored colonies resulting from analysis of mitotic recombination in AMC20.**

In the mitotic recombination experiments described in the text (data in Table 1 and Supp. Table 1), colonies that have Trp^+^/Trp^-^ sectors, but do not sector for the distal *URA3* marker represent a number of classes of intrachromatid or unequal-sister chromatid events (Fig. 2E and 2F). In intrachromatid events, the cells in the Trp^+^ sector have one *TRP1* insertion (Fig. 2E). To distinguish these classes, we treated agarose-embedded genomic DNA from the Trp^+^ side of the sectored colony with *Ngo*MIV, which does not cut within the rDNA but does cut within the *TRP1* insertion. For Trp^+^ cells resulting from intrachromatid events, we expect that *Ngo*MIV treatment will divide the rDNA gene cluster into two segments. For Trp^+^ cells resulting from unequal sister-chromatid events, we expect that *Ngo*MIV treatment will divide the rDNA gene cluster into three segments. If the unequal sister-chromatid exchange occurs between arrays that are only misaligned by a few repeats, we expect that the segment representing the region between the two *TRP1* genes will be the smallest of the three.

We examined nine Trp+ sectors derived from the *GAL-POL1* strain grown in low

levels of galactose. Six of these had three segments that hybridized to an rDNA probe.

The sizes of the smallest segments varied from about 10 kb to about 30 kb; these small

segments also hybridized to a *TRP1* probe. This result suggests that the colonies in that

have a Trp+/Trp- sector, but do not sector for the *URA3* gene usually reflect unequal

sister-chromatid recombination events in which the misalignment is usually three repeats

or less. We cannot exclude the possibility, however, that the Trp+ cells that have three

rDNA segments have one *TRP1* insertion in the rDNA, and one extrachromosomal

plasmid that contains rDNA and a copy of the *TRP1* gene; such a plasmid could be

formed by intrachromatid crossing over.

**Chromatin immunoprecipitation (ChIP) and real-time PCR analyses**

The level of Sir2p in the diploid strains AMC45 (*POL1*) and AMC20 (*GAL-POL1*) was analyzed by ChIP. Logarithmically-growing cultures or meiotic cultures (sporulated for 14 hours) were treated with formaldehyde to cross-link proteins to DNA, then Sir2p-bound DNA was immunoprecipitated with a Sir2p-directed antibody (Santa Cruz Biotechnology) by standard methods [2]. Diploid strains AMC193 (*POL1*) and AMC194 (*GAL-POL1*) were used for chromatin immunoprecipitation of Spo11p-bound DNA. Both of these strains were homozygous for an epitope-tagged version of Spo11p (*SPO11-ZZ::K.l.URA3*) and for *sae2::HPH*. In *sae2* strains, Spo11p stays covalently attached to broken DNA ends [3]. Spo11p-bound DNA was immunoprecipitated as previously described [4]. Diploid strains AMC166 (*POL1*) and AMC172 (*GAL-POL1*) were used for chromatin immunoprecipitation of Mcd1p-bound DNA. Both of these strains are homozygous for an epitope-tagged version of Mcd1p (*MCD1-ZZ::K.l.URA3*). Logarithmically growing cultures of AMC166 and AMC172 were treated with formaldehyde, then Mcd1p-bound DNA was immunoprecipitated as above. Strain constructions and complete genotypes for all strains are detailed in Supporting Tables 3 and 4.

For real-time PCR, primers for a control locus and the locus of interest were used for amplification of input DNA (for a standard curve) and of immunoprecipitated samples. Data reported are the average of two independent immunoprecipitations, each analyzed in two or three PCR reactions. Primers used for real-time PCR are described in Supporting Table 5.

**Method of DNA extraction and digestion in agarose plugs**

Cells were grown in liquid culture. 5 x 10^7^ yeast cells were suspended in 160 µl of 0.5% low-melting agarose prepared in 0.1M EDTA pH 7.5 (cells/ml), plus 0.1 mg zymolyase 20T. Agarose plugs were solidified at 4°C for 15 min and then transferred to 0.5M EDTA, 10mM Tris, pH 7.5. Plugs were incubated 5 hr to overnight at 37°C. Proteinase K and sarcosyl were added to a final concentration of 1mg/ml and 1%, respectively, and plugs were incubated for 5 hr to overnight at 50°C.

For restriction enzyme digestion of agarose-embedded DNA, plugs were washed twice for 15 min in 20mM Tris, 50mM EDTA pH 8.0 (T_20_E_50_) at 4°C, and then incubated 2 hr in 1mM PMSF in T_20_E_50_ at room temperature. Plugs were washed twice for 2 hr in T_20_E_50_ at room temperature, then soaked overnight at 4°C in TE buffer. After soaking, plugs were equilibrated for 20 min in the appropriate restriction enzyme buffer, then removed to a new tube with fresh restriction enzyme buffer plus 50-100 units of enzyme. Plugs were left overnight at the appropriate temperature for digestion. Before gel electrophoresis, plugs were soaked for 30 min in running buffer.

**Supporting Results:**

**Low Pol1p disproportionably affects the migration of chromosome XII in CHEF gels**

Although a low level of Pol1p likely impedes replication of all yeast chromosomes, given the increased rDNA instability in these cells, we investigated whether chromosome XII experiences a greater replication delay than other chromosomes under these conditions. DNA from cells in early log-phase growth was prepared in agarose blocks for CHEF gel separation of chromosomes. We observed that chromosome XII in our cells with low Pol1p did not enter the gel as efficiently as in wild-type cells, yet all other chromosomes (including the next largest, chromosome IV), migrated normally into the gel (Supp. Figure 1A). Compared to chromosome IV, the amount of XII entering the gel in wild-type cells is nearly double that of low Pol1p cells. Southern blotting of this gel with an rDNA-specific probe indicates that the missing chromosome XII DNA is not present elsewhere in the gel, suggesting that it is unable to migrate out of the well (Supp. Figure 1B). This altered migration of chromosome XII suggests that there are more stalled replication forks as a result of low cellular Pol1p.

**Cohesin binding in the rDNA is unchanged in cells with low Pol1p**

Cohesin Mcd1p binds in the non-transcribed spacer region II (NTS2) within each rDNA gene through an interaction with Lrs4p and Csm1p, preventing movement of the sister chromatids relative to each other [5,6]. This restriction of movement by cohesin effectively promotes DSBs within the rDNA to be preferentially repaired off a correctly-aligned sister chromatid, while suppressing unequal sister-chromatid exchange and crossovers between homologues. As DNA replication and the recruitment of cohesin are likely to be tightly coordinated, we considered the possibility low levels of Pol1p might impede efficient recruitment of Mcd1p to the rDNA. To analyze the level of cohesin binding in the rDNA array, we used an epitope-tagged version of Mcd1p for chromatin immunoprecipitation in wild-type cells and cells with low Pol1p. Real-time PCR was used to analyze the relative abundance of rDNA and control sequences in the immunoprecipitate. In wild-type cells and low Pol1p cells, we observed approximately equal cohesin binding within the rDNA array relative to *HIS4* binding (data not shown). As a control, we also analyzed Mcd1p binding at the cohesin-associated region 1 on chromosome III (*CARC1*) [7] and again found approximately equal levels of binding in wild-type cells and low Pol1p cells (relative to *HIS4* binding) (data not shown).

**References**

1. Kokoska RJ, Stefanovic L, DeMai J, Petes TD (2000) Increased rates of genomic deletions generated by mutations in the yeast gene encoding DNA polymerase delta or by decreases in the cellular levels of DNA polymerase delta. Mol Cell Biol 20: 7490-7504.

2. Mieczkowski PA, Dominska M, Buck MJ, Gerton JL, Lieb JD, et al. (2006) Global analysis of the relationship between the binding of the Bas1p transcription factor and meiosis-specific double-strand DNA breaks in Saccharomyces cerevisiae. Mol Cell Biol 26: 1014-1027.

3. McKee AH, Kleckner N (1997) A general method for identifying recessive diploid-specific mutations in Saccharomyces cerevisiae, its application to the isolation of mutants blocked at intermediate stages of meiotic prophase and characterization of a new gene SAE2. Genetics 146: 797-816.

4. Mieczkowski PA, Dominska M, Buck MJ, Lieb JD, Petes TD (2007) Loss of a histone deacetylase dramatically alters the genomic distribution of Spo11p-catalyzed DNA breaks in Saccharomyces cerevisiae. Proc Natl Acad Sci U S A 104: 3955-3960.

5. Huang J, Moazed D (2003) Association of the RENT complex with nontranscribed and coding regions of rDNA and a regional requirement for the replication fork block protein Fob1 in rDNA silencing. Genes Dev 17: 2162-2176.

6. Huang J, Brito IL, Villen J, Gygi SP, Amon A, et al. (2006) Inhibition of homologous recombination by a cohesin-associated clamp complex recruited to the rDNA recombination enhancer. Genes Dev 20: 2887-2901.

7. Laloraya S, Guacci V, Koshland D (2000) Chromosomal addresses of the cohesin component Mcd1p. J Cell Biol 151: 1047-1056.

8. Lemoine FJ, Degtyareva NP, Lobachev K, Petes TD (2005) Chromosomal translocations in yeast induced by low levels of DNA polymerase a model for chromosome fragile sites. Cell 120: 587-598.

9. Goldstein AL, McCusker JH (1999) Three new dominant drug resistance cassettes for gene disruption in Saccharomyces cerevisiae. Yeast 15: 1541-1553.

10. Stapleton A, Petes TD (1991) The Tn3 beta-lactamase gene acts as a hotspot for meiotic recombination in yeast. Genetics 127: 39-51.

11. Storici F, Lewis LK, Resnick MA (2001) In vivo site-directed mutagenesis using oligonucleotides. Nat Biotechnol 19: 773-776.

12. Puig O, Rutz B, Luukkonen BG, Kandels-Lewis S, Bragado-Nilsson E, et al. (1998) New constructs and strategies for efficient PCR-based gene manipulations in yeast. Yeast 14: 1139-1146.
